# Extreme Weather, Vulnerable Populations, and Mental Health: The Timely Role of AI Interventions

**DOI:** 10.3390/ijerph22040602

**Published:** 2025-04-11

**Authors:** Mehak Batra, Bircan Erbas

**Affiliations:** Department of Public Health, School of Psychology and Public Health, La Trobe University, Melbourne, VIC 3086, Australia; m.batra@latrobe.edu.au

**Keywords:** environmental disasters, mental health, climate change, artificial intelligence, intersectionality

## Abstract

Environmental disasters are becoming increasingly frequent and severe, disproportionately impacting vulnerable populations who face compounded risks due to intersectional factors such as gender, socioeconomic status, rural residence, and cultural identity. These events exacerbate mental health challenges, including post-traumatic stress disorder (PTSD), anxiety, and depression, particularly in low- and middle-income countries (LMICs) and underserved areas of high-income countries (HICs). Addressing these disparities necessitates inclusive, culturally competent, intersectional, and cost-effective strategies. Artificial intelligence (AI) presents transformative potential for delivering scalable and culturally tailored mental health interventions that account for these vulnerabilities. This perspective highlights the importance of co-designing AI tools with at-risk populations, integrating these solutions into disaster management frameworks, and ensuring their sustainability through research, training, and policy support. By embedding mental health resilience into climate adaptation strategies, stakeholders can foster equitable recovery and reduce the long-term mental health burden of environmental disasters.

## 1. Introduction

As scientists examining the interplay between climate change and public health, we are deeply concerned about the increasing frequency and severity of environmental disasters [1]. Events in 2023, such as unprecedented heatwaves across Europe, devastating floods in Pakistan, and record-breaking wildfires in Canada, have starkly brought to our attention that these crises are no longer theoretical projections of a faraway future but are pressing challenges of our present. The World Meteorological Organization named 2023 as the hottest year on record, further marking a critical inflection point in this global crisis [2].

Warmer temperatures drive more frequent and extreme heatwaves, excessive precipitation, droughts, and fires, with severe consequences for human populations. As climate change intensifies, its impacts are not felt equally across all communities. Human vulnerability to environmental disasters include exposure, sensitivity, and adaptive capacity [3]. Exposure refers to physical risks based on geography, such as coastal communities facing rising sea levels or urban areas experiencing extreme heat and bushfires. Sensitivity describes how social, cultural, and economic factors—like income, health status, and infrastructure—affect a population’s susceptibility to disasters. Adaptive capacity is how individuals cope with and recover from such events, influenced by local systems, governance, education, resource availability, and community collegiality. Vulnerability varies by population density, socioeconomic status, and systemic inequities. For example, low-income urban neighbourhoods face greater heat exposure due to poor housing and a lack of funds for heat relief, while rural populations often experience delays in emergency responses. Understanding these dynamics is essential for designing targeted, equitable interventions.

Among the most devastating impacts are floods, which displace millions of residents, destroy homes, and disrupt access to essential resources, including clean water and healthcare. These events particularly affect marginalised populations, who face heightened risks of injury, disease, and long-term displacement due to pre-existing vulnerabilities. Although less directly impactful for certain populations, heatwaves exacerbate existing health conditions and strain already fragile healthcare systems. Environmental disasters also amplify respiratory health threats, such as asthma, by altering atmospheric pressure and allergen exposure. For instance, increased CO_2_ levels and warming temperatures extend pollen seasons and worsen allergic respiratory conditions [4]. One notable phenomenon, thunderstorm asthma, occurs when storms break pollen grains into smaller particles, triggering acute asthma attacks during peak seasons [5]. This highlights how extreme weather events exacerbate public health crises, particularly for disadvantaged groups who are least equipped to adapt to economic, geographic, and systemic barriers. When these events intersect with systemic inequities, they become disasters, amplifying destruction and deepening mental health and well-being crises in affected populations.

It has been documented that environmental disasters can have direct impacts, such as trauma and anxiety due to extreme weather conditions, and indirect impacts, including heat-related illnesses. The dislocation of built and physical environments disrupts social structures, further exacerbating mental health challenges. Studies have consistently shown that extreme weather events such as floods, storms, and wildfires can lead to post-traumatic stress disorder (PTSD), depression, and anxiety [6]. Globally, PTSD rates vary depending on the disaster type, healthcare access, and socioeconomic factors. In the U.K., prevalence rates within 12 months of extreme weather events were 19.8% for anxiety, 21.35% for depression, and 30.36% for PTSD [7]. The 2019–2020 Black Summer bushfires in Australia led to probable PTSD in 14% to 17.5% of directly affected individuals and 28% to 47.6% of firefighters and emergency responders [8]. Studies of Hurricane Katrina (2005) survivors in the United States show a varying prevalence. Research focusing on vulnerable populations, such as low-income parents in New Orleans, reported nearly half of respondents exhibiting probable PTSD [9]. In Bangladesh, PTSD was reported in 48.1% of child and adolescent survivors of Cyclone Sidr (2007) [10], while in Haiti, the 2010 earthquake left 28.4% of survivors with PTSD symptoms [11].

Vulnerable populations, such as those living in poverty, farmers, and children, are particularly at risk, with the incidence and prevalence of mental health issues expected to rise as environmental disasters and extreme weather events become more frequent and severe. This also includes culturally and linguistically diverse (CALD) communities and migrants/refugees, as these groups often carry pre-existing trauma from displacement, conflict, or other adverse experiences, which the impacts of extreme weather events can exacerbate. Furthermore, language barriers, cultural differences in mental health understanding, and limited access to culturally appropriate support services can significantly hinder their ability to cope and recover. The recent devastating floods in Shepparton, Melbourne, Australia, are a stark reminder that these vulnerabilities are not confined to specific regions; they are present within communities with a substantial proportion of already displaced and vulnerable individuals and require targeted support and intervention [12].

New and emerging phenomena, such as eco-anxiety and climate grief, are increasingly common alongside immediate psychological responses [13]. These prolonged emotional and psychological impacts can contribute to conditions like PTSD, particularly when coupled with the ongoing and potentially worsening effects of climate change. Young people are commonly afflicted with existential fears of inheriting a fragile and uncertain future, furthered by pervasive media coverage and social pressures to act.

Artificial intelligence (AI) is increasingly being integrated into disaster response and recovery to enhance access to mental health support, particularly in underserved and crisis-affected communities. In the United States, the Crisis Text Line uses AI to triage millions of text messages during disasters such as hurricanes, wildfires, and mass shootings, identifying individuals at high risk of self-harm and directing them to trained human responders. With over six million users, the platform has shown promising results in reducing immediate psychological distress, particularly in rural and remote areas where in-person support is limited [14]. Similarly, Wysa, an AI-powered chatbot offering cognitive behavioural therapy (CBT)-based support, was widely adopted during the COVID-19 pandemic across countries like India, the U.K., and the U.S. It provides anonymous, 24/7 psychological support to users experiencing anxiety and depression, especially in low-resource settings, and early evaluations have demonstrated improvements in mental health outcomes [15]. These tools illustrate the potential of AI-driven interventions to deliver timely, scalable, and culturally adaptable mental health care in disaster contexts.

This perspective aims to highlight the profound mental health impacts of environmental disasters, particularly on vulnerable populations, including those in low- and middle-income countries (LMICs) and underserved areas of high-income countries (HICs). It emphasises the transformative potential of AI in delivering scalable, culturally tailored mental health interventions. By integrating AI tools into disaster management frameworks and addressing the specific needs of LMICs—such as limited infrastructure and digital literacy—this perspective seeks to foster equitable recovery, reduce systemic inequities, and mitigate the long-term psychological burden of environmental crises.

## 2. Global Disparities in the Mental Health Impacts of Environmental Disasters

The mental health impacts of environmental disasters are more apparent in LMICs due to poverty, low education levels, and social exclusion, that elevate vulnerability [16]. Similarly, in HICs, including Australia, individuals living in rural and remote areas or less-advantaged areas face disproportionate impacts, often due to limited access to healthcare, social services, and emergency resources. While much of the research and policy attention has focused on HICs, regions such as sub-Saharan Africa, South Asia, and small island developing states (SIDS) face unique and severe challenges, yet they often remain underrepresented in global discourse. While these disparities are widespread, their impact varies significantly across regions based on socioeconomic conditions, governance, and healthcare accessibility. Below, we examine how disaster-affected populations in sub-Saharan Africa and South Asia experience compounding risks, with AI emerging as a potential tool to address some of these gaps.

### 2.1. Disparities in Disaster Response and Recovery

In sub-Saharan Africa, drought and flooding have displaced millions of people, particularly in fragile states such as Somalia and Sudan. The most recent floods in Sudan in 2022 displaced hundreds of thousands of people, exacerbating their already precarious mental health situation due to decades of trauma from fighting and food insecurity. Despite this scale of crises, systems for psychological support remain extremely minimal within the region [17]. Long-term displacement in such areas generates a cycle of continuous stress and insecurity that puts survivors in a state of psychological limbo.

Similarly, in South Asia, Bangladesh, India, and Pakistan are highly densely populated nations that are often at the receiving end of cyclones and monsoons. Whole communities get swept out, with survivors being crowded into under-resourced refugee camps where stress levels surge. The cultural stigma surrounding mental health in these regions further compounds the problem, silencing those in need. For instance, in the aftermath of Cyclone Amphan in 2020, mental health professionals in Bangladesh reported sharp increases in anxiety and depression cases, yet many of the affected individuals were unwilling to seek help [18]. AI-driven mental health apps tailored to regional languages and cultural contexts could help bridge these gaps by offering anonymous and accessible psychological support.

These narratives emphasise the critical importance of addressing mental health during disaster recovery. Cultural and structural changes at the community level, codesigned and driven by individuals within these communities, are essential to enable close and continuous uptake monitoring over time. This approach is one way to ensure that adaptive interventions for recovery and improvements in mental health and well-being remain sustainable in the long term. AI has already demonstrated its potential to address disparities by improving access to culturally sensitive, affordable, and scalable mental health interventions. However, its application in disaster-affected regions remains underutilised. Below, we highlight key AI-driven solutions that have successfully enhanced disaster response and mental health support in resource-limited settings.


AI Solutions in Disaster-Affected RegionsAI Chatbots (Wysa, Woebot)
Used in India during the COVID-19 pandemic to support frontline workers and isolated individuals with stigma-free, 24/7 mental health care [19].Multilingual Mental Health Apps (Tarjimly) During the 2021 Afghan refugee crisis, the Tarjimly app provided real-time, AI-assisted interpretation in languages like Dari and Pashto, facilitating communication for refugees and aid workers in regions such as Spokane, Washington, DC, USA [20].Screening Bots (Tess) Deployed in Texas, USA, after Hurricane Harvey (2017) to detect PTSD and anxiety in displaced communities via SMS/chat.Predictive AI (Minderoo Foundation) Applied in Australia following the Black Summer bushfires (2019–20) to map mental health risks and guide rural resource allocationSocial Media Monitoring Used during the Kerala floods (India, 2018) to identify distress signals online and direct aid to remote communities [21].AI Telehealth Enabled remote mental health care in Puerto Rico after Hurricane Maria (2017) when clinics were inaccessible [22].AI: artificial intelligence, PTSD: post-traumatic stress disorder, NGO: non-Government organisations.


### 2.2. Economic Inequalities

Inequalities in resource utilisation and systemic responses between LMICs and HICs further heighten these differences. In HICs, better healthcare and superior psychological aid enable faster and higher recovery levels. For example, following the destruction caused by Hurricane Katrina in the United States, trauma-focused cognitive behavioural therapy and organised mental health programs played a critical role in supporting survivors [9]. However, these initiatives were often short-term, reliant on temporary federal funding, and lacked sustainability as they were difficult to monitor. Integrating mental health services into existing systems and training local professionals in consultation with impacted community members could ensure long-term resilience in disaster-affected populations.

The economic inequalities further outline the disparities in the financial burden LMICs face. According to the World Bank, the annual average cost of climate-related disasters in LMICs was $29 billion—a significant proportion of their GDP [23]. Such economic shocks further burden already limited resources and constrain investment in either mental health resilience or a holistic recovery process. Meanwhile, the United States experienced over $145 billion in damages due to weather-related disasters in 2021; however, the long-term economic impacts were mitigated by robust U.S. recovery mechanisms such as disaster insurance and federal aid [24].

Most disaster recovery efforts in LMICs focus on immediate physical needs, such as food, shelter, and infrastructure, while mental health support is less emphasised. This creates a significant imbalance with serious long-term cost implications. Untreated mental health conditions contribute to lower productivity, higher healthcare costs, and perpetuating cycles of poverty. For example, studies on flood victims in Bangladesh reveal heightened rates of anxiety, depression, and PTSD [10]. While some demonstrate remarkable resilience through community support networks, the lack of structured mental health interventions often leaves survivors struggling to rebuild their lives [25].

AI can help bridge economic disparities by reducing the costs of delivering mental health interventions. AI-based teletherapy services can provide affordable, scalable mental health care, especially for populations that cannot access in-person treatment. Additionally, AI-driven predictive models can assess economic vulnerabilities and allocate resources more efficiently, helping governments and aid organisations in targeting the most-needed interventions.

### 2.3. Funds Available and Governance Challenges

In many LMICs, governance challenges and limited resources create inefficiencies in disaster response and mental health support. Weak infrastructure for fund allocation and management often results in resources failing to reach the communities that need them most. Often, governments with already limited infrastructure and resources are unprepared for the scale and complexity of new and unexpected disasters, leaving local councils and the communities that live in the affected areas without the necessary funding or resources. Preparedness at the government level incorporates the voice of the lived experience of community members and plays a crucial role in ensuring that systems are in place for effective disaster response. This preparedness includes adequate training for government officials, streamlined coordination across agencies, and funding availability for local councils to build community resilience that is sustainable over time.

By contrast, HICs with stable political systems are better equipped to respond effectively to disasters. For instance, countries such as Japan have integrated disaster management systems that include mental health care in response to disasters [26]. These systems depend on clear protocols, adequate funding, and intersectoral collaboration to provide psychological interventions alongside physical recovery efforts. Incorporating such approaches globally, emphasising governance preparedness and equitable funding distribution, is critical to building effective and resilient disaster response systems.

### 2.4. Global Climate Responsibility

Mental health disparities from environmental disasters present profound ethical challenges. Despite contributing less than 4% of global CO2 emissions, countries in sub-Saharan Africa have experienced severe climate impacts for decades, including droughts, floods, and extreme heat [27]. These events have displaced millions of people and taken a significant psychological toll, with studies showing that 50% of flood-displaced individuals in rural areas of sub-Saharan Africa report PTSD, anxiety, or depression [28]. HICs, responsible for approximately 70% of historical emissions, have stronger recovery systems and face fewer challenges, emphasising the responsibility to address these inequities by supporting strategies to develop and incorporate mental health interventions and climate resilience efforts in vulnerable regions.

From the climate justice perspective, HICs, as major contributors to global emissions, have a moral responsibility to provide financial, technical, and policy support to mitigate mental health impacts in LMICs. Programs like the Green Climate Fund (GCF) have been established to channel resources for climate adaptation and resilience [29]. As of 2024, the GCF has committed $13.5 billion across 243 projects in 129 countries, including both low- and middle-income nations as well as some high-income countries like Australia. While primarily being focused on mitigation and adaptation efforts, fund allocation tends to prioritise physical infrastructure, with limited investment in psychological well-being and mental health support [30]. Integrating mental health resilience into international climate funding frameworks is necessary to address this imbalance. Such an approach would ensure not only immediate support for vulnerable populations, but also address the profound and intergenerational effects of climate change, fostering equitable and sustainable recovery in LMICs. AI can be a key tool in global climate resilience efforts. AI-powered climate forecasting models help to predict and mitigate disaster impacts, while AI-driven mental health applications offer scalable support to affected populations. Integrating mental health resilience into international climate funding frameworks is necessary to address this imbalance.

Forced migration due to environmental disasters can lead to significant mental health challenges. Displacement caused by extreme weather, including flooding and prolonged droughts, results in a loss of identity, social connections, and cultural heritage, compounded by the stress of adapting to new environments. In LMICs, migration is often internal or regional, with the limited resources in receiving areas leading to overcrowding, inadequate mental health care, and heightened social tension. For example, Cyclone Amphan in 2020 displaced 2.4 million people in Bangladesh and India, leaving many in urban slums or camps with little access to mental health services, resulting in heightened anxiety, depression, and hopelessness [18]. In contrast, migrants in HICs face systemic barriers such as restrictive immigration policies, discrimination, and a lack of cultural competence in mental health care.

Addressing these disparities requires action at the global level. International funding mechanisms, such as climate adaptation funds, must prioritise psychosocial resilience alongside physical infrastructure recovery. Continuous capacity-building efforts, including training local mental health professionals, are essential for establishing sustainable support systems. Culturally tailored interventions are also crucial, as strategies effective in HICs may not be applicable in LMIC contexts. Incorporating intersectionality into global initiatives ensures equitable resource distribution and responsiveness to the diverse needs of vulnerable and intersecting populations. This approach strengthens local capacities while aligning global efforts with the lived realities of those who are most affected.

## 3. Intersectionality and the Mental Health Impacts of Environmental Disasters

Intersectionality offers a valuable perspective for examining how interconnected vulnerabilities—such as biological sex or being female and from underserved populations, socioeconomic status, disability, cultural identity, and rural residence—further contribute to the long-term detriment of environmental disasters’ mental health impacts. These intersecting factors create multiple layers of disadvantage, especially amongst those who have lifelong exposure to situations of vulnerability. Thereby, leading to continual disparities in exposure to risk, the adequacy of response efforts, and the ability to recover effectively.

Environmental disasters particularly heighten inequities disproportionately affecting women. As primary caregivers, women often face increased emotional and physical burdens during crises. Many women from rural/remote areas and lower social and financial positions lack language or financial literacy, exacerbating problems during environmental degradation. For instance, in rural South Asia, women affected by floods and droughts reported significantly higher levels of anxiety and depression due to the combined pressures of caregiving and resource scarcity [31]. Furthermore, displacement into overcrowded and unsafe shelters exacerbates these challenges, increasing the risk of gender-based violence (GBV) and adding another layer of trauma. Similarly, research following Hurricane Katrina revealed that women, particularly those with children, were disproportionately affected, with elevated rates of PTSD and depression linked to caregiving stress and financial insecurity [9].

The mental health burden increases manifold due to poverty, which limits access to resources, safe housing, and healthcare. Low-income individuals are more likely to live in high-risk areas such as floodplains or poorly constructed housing and face barriers to evacuation. These individuals often lack insurance or savings, leading to prolonged financial insecurity and recovery, which exacerbates chronic stress, depression, and anxiety. For instance, the 2022 floods in Lismore, Australia, left many older women homeless, many of whom were already financially vulnerable. This highlights how disasters disproportionately impact at-risk populations and exacerbate mental health challenges [32]. Similarly, survivors of Hurricane Katrina without financial safety nets reported significantly higher rates of PTSD compared to their wealthier counterparts [9].

Rural populations face specific challenges during environmental disasters. Rural areas typically have fewer healthcare facilities, resulting in limited access to mental health services. These regions experience longer delays in receiving aid and are particularly vulnerable to bushfires and droughts. For instance, the Australian bushfires had severe mental health impacts, including eco-anxiety and PTSD, especially among farmers and rural communities dependent on the land for their livelihoods. Thunderstorm asthma events, caused by high pollen levels during storms, also disproportionately affect rural populations, where healthcare responses are slower and asthma management resources are limited [33]. Recurring floods in Australia have similarly devastated rural and remote communities, displaced families, and for most, destroyed homes, farms, and livelihoods—often without adequate disaster insurance or rebuilding resources. This prolonged financial and psychological distress underscores the need for targeted interventions that address both economic recovery and mental health in rural and remote areas, even in high-income countries.

Social networks play a vital role in recovery from environmental disasters. In rural LMIC settings, informal community networks often serve as survivors’ primary source of support. While these networks are resilient, they are typically under-resourced—in terms of staff trained in providing mental health support and financial support, but also in terms of the built environment sometimes making it difficult to get support to rural, remote areas and being unable to address the scale of mental health challenges caused by repeated environmental disasters. For example, in rural Bangladesh, local communities often depend on informal support systems such as neighbours and extended family to cope with displacement and trauma [34]. While invaluable, these networks cannot substitute for formal psychological interventions. In contrast, urban HIC settings utilise formal institutions such as hospitals, social services, and non-government organisations to provide comprehensive support. These systems are better equipped to coordinate large-scale mental health responses, offering trauma counselling and ensuring access to necessary medications.

People with disabilities also face specific challenges during environmental disasters. Barriers to mobility, sensory impairments, or cognitive disabilities can make evacuation difficult, leaving them disproportionately vulnerable to harm. Studies have shown that individuals with disabilities are more than twice as likely to develop PTSD following disasters due to physical vulnerabilities and neglect in recovery processes [32]. Furthermore, the lack of inclusive mental health services adds to feelings of isolation and abandonment, compounding their psychological distress.

Cultural identity has a significant influence on mental health outcomes. During disaster events, Indigenous groups and culturally marginalised populations often experience cultural trauma when sacred places are destroyed, or traditional practices are disrupted. For instance, the loss of ancestral lands during Australian bushfires has caused profound grief and eco-anxiety among Aboriginal communities [35]. Similarly, migrants and refugees face unique mental health challenges during disasters, as these events often exacerbate the trauma they fled from in the past, compounding fears for their future. Despite these challenges, interestingly, migrants and refugees frequently display remarkable resilience in the face of such disasters, leveraging strong social bonds and adaptive coping mechanisms. Systematic discrimination and language barriers, however, further limit their access to mental health resources and recovery programs, highlighting the need for culturally sensitive interventions [36].

AI-driven mental health interventions do offer potential solutions for disaster contexts but also present challenges that may exacerbate existing disparities. Infrastructure damage and the digital divide disproportionately affect rural, low-income, and marginalised groups, limiting access to AI-based mental health services. Additionally, algorithmic bias in AI models, which are often trained on Western data, can misinterpret distress symptoms in culturally diverse populations, reducing their effectiveness. Language barriers and literacy gaps further hinder accessibility. AI interventions must incorporate inclusive datasets, culturally adaptive models, and human oversight to address these challenges to ensure equitable, contextually relevant mental health support. AI risks reinforcing rather than alleviating disaster-related mental health inequities without such adaptations.

**Figure 1:**
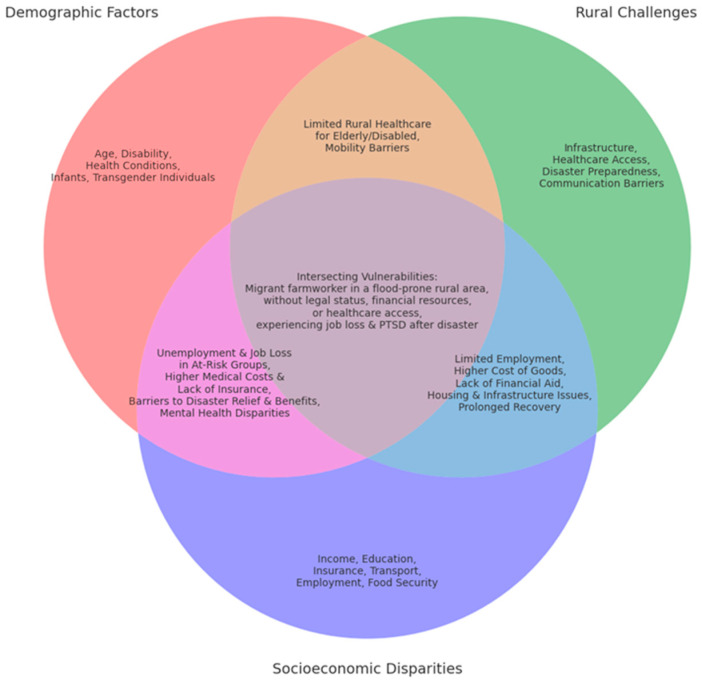
Intersectionality in Mental Health Impacts of Environmental Disasters.

These cumulative vulnerabilities, often across the life course, create severe disparities in mental health outcomes. A low-income woman with a disability living in a flood zone may face difficulty evacuating, increased stress from caregiving responsibilities, and financial insecurity, all of which heighten the impacts of disasters. Similarly, a rural resident or elderly Indigenous individual displaced by wildfires may confront cultural trauma, physical vulnerabilities due to age, and exclusion from recovery resources. The figure (Figure 1) illustrating the intersection of gender inequity, rural challenges, and poverty highlights how these compounded vulnerabilities disproportionately impact groups such as women in rural areas and rural poor populations. For example, the 2022 Lismore floods saw older women in rural areas, who were already economically disadvantaged, losing homes and livelihoods, leading to prolonged homelessness and mental health challenges [37]. This case study underlines how intersecting vulnerabilities manifest during disasters, emphasising the urgent need for targeted, inclusive mental health interventions that address these overlapping dimensions of vulnerability and promote equitable recovery.

## 4. Call for Inclusive and Targeted Interventions with Artificial Intelligence (AI) Integration

The accelerating mental health impacts of environmental disasters necessitate the adoption of inclusive, culturally competent, cost-effective, and intersectional strategies. These approaches are important to address the diverse vulnerabilities among affected populations and support a sustainable and equitable pathway to recovery. Culturally sensitive programs can bridge critical gaps, such as training community health workers in psychological first aid [38] and integrating Indigenous practices [39]. Addressing intersectional vulnerabilities is necessary for effective interventions. Women, rural residents, people with disabilities, and indigenous populations face unique challenges during environmental disasters. Gender-sensitive interventions, inclusive evacuation routes, and culturally adapted mental health resources will support pathways to recovery. Enhancing healthcare infrastructure, telemedicine, and mobile clinics for rural areas can mitigate delays in receiving mental health support [40].

While culturally tailored mental health programs are essential, their scalability and accessibility remain limited in many disaster-affected regions. AI offers a transformative approach by augmenting these interventions with predictive analytics, virtual support systems, and real-time crisis response tools. These technologies can integrate culturally tailored mental health resources to address the unique needs of vulnerable groups, including women, rural residents, people with disabilities, and Indigenous populations.

For instance, predictive analytics, such as Google’s Flood Hub, can prioritise high-risk areas and vulnerable groups by analysing satellite and historical disaster data. AI-driven chatbots, such as Wysa and Woebot, provide immediate, multilingual support even in resource-constrained settings, offering therapeutic conversations based on cognitive behavioural therapy (CBT) principles [41,42]. Programs such as the Scalable Person-Centered Interventions for Resilience through Integrated Technologies (SPIRIT) exemplify this by leveraging AI to provide culturally relevant mental health resources and resilience training, equipping local leaders to deliver psychological first aid. Offline functionalities ensure that these tools remain effective in disaster-hit regions with limited connectivity. Additionally, AI-powered mental health triage systems, such as Crisis Text Line, analyse real-time language patterns to assess crisis severity and prioritise high-risk individuals for human intervention. AI can also enable real-time monitoring through social media and wearable data, allowing the early identification of mental health crises and optimising resource distribution to underserved areas [43]. AI-driven tools such as machine learning algorithms deployed on social media platforms like Twitter and Facebook have been used to detect distress signals, suicide risks, and PTSD-related expressions, facilitating timely interventions.

An innovative tool that can adapt language, context, and other user-enabled facets is the Ecological Validity Model (EVM), which offers a culturally responsive framework for shaping mental health interventions [44]. For example, the EVM has been applied in adapting therapies for Latino populations in the United States, demonstrating a strong uptake and cultural appropriateness [45]. AI tools guided by the EVM can adjust for key dimensions such as culturally informed mental health beliefs, idioms of distress, and communication norms. In healthcare settings, Google Translate has assisted clinicians in communicating with patients from linguistically diverse backgrounds, including in mental health contexts. Although studies have shown that it improves accessibility, especially where interpreters are unavailable, concerns remain about its accuracy in conveying complex psychological terminology [46]. To address these limitations, human–AI collaboration involving bilingual clinicians or cultural mediators is critical in validating AI outputs and reducing potential biases [47]. In disaster-affected rural areas, AI mental health chatbots such as Tess have provided immediate psychological support, offer coping strategies, and they connect individuals with local mental health services when available [48]. AI models are also being trained to recognise culturally specific expressions of psychological distress, such as somatic symptoms common in South Asian communities, although their accuracy remains variable and complex cases still require human oversight. Additionally, AI-assisted mental health triage systems have been piloted in resource-limited settings like refugee camps in Jordan and post-disaster areas in Haiti, showing promise in improving screening and referral efficiency [49]. These examples highlight how culturally adapted, AI-enhanced tools can support more equitable mental health care in diverse and crisis-affected populations when embedded within frameworks like the EVM.

Many disaster recovery efforts fail to account for the layered and intersecting vulnerabilities such as socioeconomic disadvantage, disability, and cultural and linguistic diversity that influence mental health outcomes. AI can help bridge this gap by enabling real-time, tailored interventions for the most at-risk populations. For example, during the 2019–2020 Black Summer bushfires in Australia, AI-powered systems were used to map fire risk zones and predict the spread using real-time satellite and environmental data [50]. Building on these projects, like with the Minderoo Foundation’s Fire and Flood Resilience Initiative, we have begun integrating social and health vulnerability indices to better inform response planning and community outreach [51]. By layering geospatial fire data with information on income levels, chronic illness, and mental health service availability, these systems are beginning to identify which communities may need mental health support most urgently. Embedding intersectionality into AI design for instance, by incorporating CALD status, age, and disability would further strengthen the ability of these tools to address disparities and enhance post-disaster mental health outcomes.

Despite AI’s potential to bridge mental health disparities, its implementation is not uniform across all settings. While HICs have the necessary digital infrastructure, LMICs often struggle with limited resources, digital literacy, and technological accessibility. Addressing these challenges requires innovative approaches such as offline AI tools, mobile-based interventions, and localised training programs. AI-powered offline tools, such as mobile apps and chatbots, can help bridge these gaps by providing scalable, community-based interventions that empower local leaders with data-driven insights, including multilingual information sharing. Initiatives such as the WHO’s EQUIP platform can further address these disparities by emphasising cultural competency and scalability. Through e-learning courses and digital tools, EQUIP enhances mental health training and improves service delivery in diverse and underserved settings. While EQUIP has received funding from international health organisations, its implementation relies on national adoption. Countries can integrate EQUIP into existing mental health programs by forming WHO partnerships, securing government support, and embedding it into workforce training initiatives [52].

Integrating AI-driven mental health solutions into disaster recovery frameworks ensures a holistic and equitable approach. By embedding mental health metrics into policy strategies, AI can mitigate immediate psychological impacts and build long-term resilience, particularly for marginalised and underserved communities. As AI evolves, its role in disaster management must prioritise inclusivity, ethical governance, and sustainability to prevent the deepening of existing inequalities.

**Figure 2:**
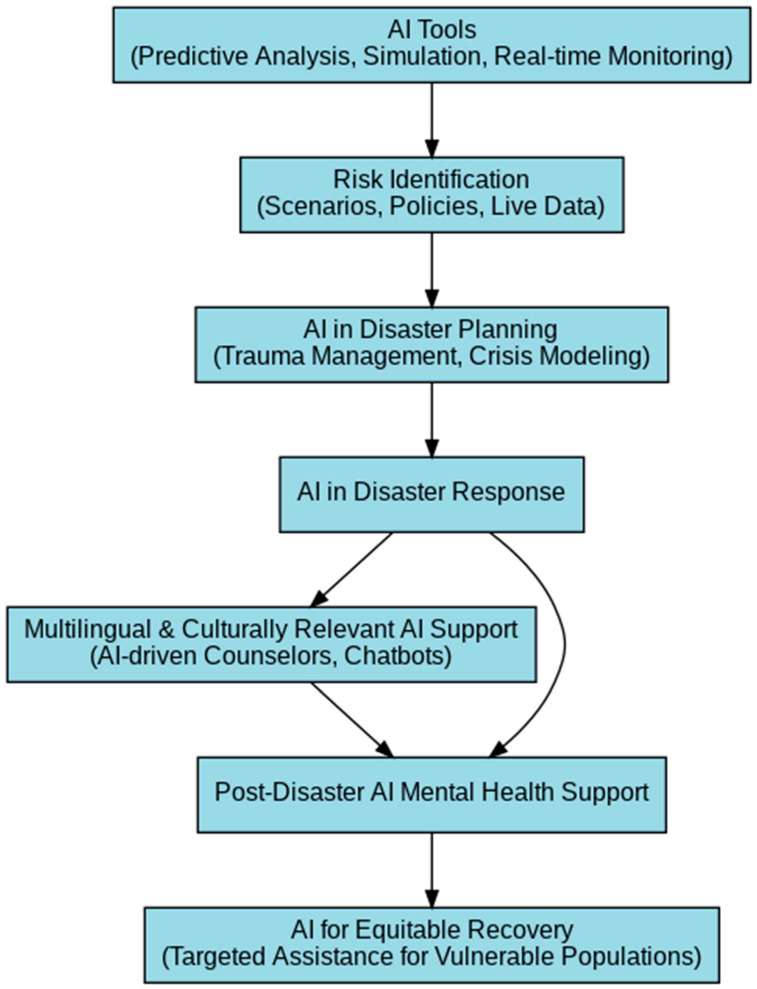
AI-Driven Framework for Supporting Mental Health in Disaster Management.

Figure 2 illustrates the role of AI-powered tools in disaster response, integrating predictive analytics, culturally tailored digital mental health interventions, and real-time monitoring. By leveraging AI-driven insights, disaster management frameworks can enhance accessibility, reduce the response time, and ensure culturally competent mental health support for vulnerable groups. The diagram highlights AI’s role in multilingual chatbot support, predictive modelling, and digital translation tools that break barriers for linguistically diverse communities. By embedding AI into disaster mental health strategies, both immediate response and long-term recovery efforts can be optimised for sustainable impact.

## 5. Conclusions

The long-term mental health impacts of environmental disasters remain a persistent and worsening issue, disproportionately affecting vulnerable populations who are least equipped with the knowledge, infrastructure, and resources to adapt and recover. Understanding the systemic inequities that place these groups at the greatest risk is essential to developing interventions that are likely to have the greatest impact. AI offers transformative potential as a cost-effective and scalable solution, but its success depends on inclusive co-design with at-risk members of the population.

Ethical considerations are paramount in the development and deployment of these AI-driven interventions. Future research must uphold principles of justice, beneficence, and respect for autonomy by embedding community voices at all stages of design and implementation. Transparency, data privacy, and informed consent are critical, especially for populations with limited digital or health literacy. Ethical frameworks should also guide efforts to mitigate algorithmic biases, address power imbalances, and avoid exacerbating existing mental health inequities.

Future research should focus on testing the implementation of collaborative, sustainable AI-driven solutions, assessing adherence to technology, and training community members from culturally and economically diverse populations to embrace AI as a tool for effective and equitable disaster management. Additionally, research is needed to address existing gaps in AI’s ability to handle complex post-disaster mental health scenarios over a long period of time, particularly for intersectional marginalised populations. Investigating ways to mitigate algorithmic biases, improve contextual adaptability, and ensure culturally responsive AI interventions should be prioritised. Further exploration into hybrid models integrating AI with human-led mental health care could enhance intervention effectiveness, ensuring that technological solutions complement, rather than replace, essential human support systems in disaster-affected communities.

## Data Availability

No new data were created or analyzed in this study. Data sharing is not applicable to this article.
